# Thermosonication-assisted fortification of kiwi juice with bee bread: enhancing nutritional and functional properties through ANFIS-RSM optimization

**DOI:** 10.3389/fnut.2025.1657136

**Published:** 2025-08-04

**Authors:** Seydi Yıkmış, Aylin Duman Altan, Melikenur Türkol, Nazlı Tokatlı, Çiğdem Yıldırım Maviş, Nazan Tokatlı Demirok, Rana Muhammad Aadil, Emad Karrar, Moneera O. Aljobair, Isam A. Mohamed Ahmed

**Affiliations:** ^1^Department of Food Technology, Tekirdag Namık Kemal University, Tekirdag, Türkiye; ^2^Department of Industrial Engineering, Tekirdag Namık Kemal University, Tekirdağ, Türkiye; ^3^Nutrition and Dietetics, Faculty of Health Sciences, Halic University, Istanbul, Türkiye; ^4^Department of Computer Engineering, Faculty of Engineering and Natural Sciences, Istanbul Health and Technology University, Istanbul, Türkiye; ^5^Department of Nutrition and Dietetics, Faculty of Health Sciences, Tekirdag Namık Kemal University, Tekirdag, Türkiye; ^6^National Institute of Food Science and Technology, University of Agriculture, Faisalabad, Pakistan; ^7^Department of Plant Sciences, North Dakota State University, Fargo, ND, United States; ^8^Department of Sports Health, College of Sports Sciences and Physical Activity, Princess Nourah Bint Abdulrahman University, Riyadh, Saudi Arabia; ^9^Department of Food Science and Nutrition, College of Food and Agricultural Sciences, King Saud University, Riyadh, Saudi Arabia

**Keywords:** adaptive neuro-fuzzy inference, bee bread, bioactive substances, kiwi juice, response surface methodology, thermosonication

## Abstract

This study investigated the effects of thermosonication on the preservation and enhancement of bioactive components in kiwi juice fortified with bee bread. Response Surface Methodology (RSM) and Adaptive Neuro-Fuzzy Inference System (ANFIS) were employed to optimize processing parameters by evaluating FRAP, total phenolics, total chlorophyll, and ascorbic acid levels. Thermosonication significantly enhanced the levels of phenolic compounds (127.97 GAE mg/100 mL) and ascorbic acid (14.89 mg/100 mL), while a reduction in chlorophyll content was observed. The ANFIS model provided more accurate predictions compared to RSM, particularly under optimal processing conditions. Additionally, the thermosonication-treated kiwi juice with bee bread (TS-KJB) exhibited the highest antioxidant capacity, total flavonoid, and dietary fiber content. The findings demonstrate that thermosonication is an effective and sustainable technique for improving the functional and nutritional properties of bee bread-fortified kiwi juice. This approach offers a promising alternative for the production of additive- and preservative-free functional fruit juices.

## 1 Introduction

Recently developed “clean label” techniques, such as ultrasound, have been the subject of research in fresh produce processing (1–3). The ultrasound mechanism generates and transmits sound waves in the 20–100 kHz frequency range in a solvent by a piezoelectric device by converting them into electromechanical energy (4). This process causes pressure changes and the formation of bubbles within the liquid, triggering acoustic cavitation, i.e., the coalescence and collapse of bubbles (5). The cavitation phenomenon, when combined with a certain temperature, is referred to as thermosonication (TS) (6). TS, also known as ultrasonic or acoustic heating, preserves the nutritional and physiological benefits of fruits and vegetables while eliminating pathogens and spoilage micro-organisms (7, 8). Researchers have applied thermosonication to various fruit and vegetable juices such as blackberry juice (9), guava juice (10), African Mango Fruit (*Irvingia Gabonensis*) juice (11), Kutkura (*Meyna Spinosa*) juice (12), Amora (*Spondius Pinnata*) juice (13), Hog Plum (*Spondias Mombin L*.) juice (14), tangor fruit juice (15), watermelon-beetroot juice (16), watermelon juice (17), parsley juice (18).

RSM is a statistical method to optimize processes. It involves designing experiments, building models, and studying how variable changes affect the system’s response (19). The ANFIS is a neuro-fuzzy technique that combines artificial neural networks’ structural design and learning capabilities. ANFIS uses a pre-existing data set and a fuzzy inference system to analyze and interpret the learned weight parameters (20).

Kiwi (*Actinidia* species) is a temperate climate fruit native to southwest China, which has gained an important place in the international fruit trade, especially with the *Actinidia deliciosa* and *Actinidia chinensis* species. Rich in vitamin C, flavonoids, phenolic compounds, fiber, and minerals, kiwi offers excellent health benefits thanks to these nutrients (21). Kiwi is a commercially valuable fruit with a unique flavor and high nutritional content. Rich in bioactive phytochemicals, kiwi offers health benefits but, due to its climacteric structure, cannot be stored for long periods after ripening and is prone to spoilage. Kiwi products should be processed to prevent spoilage and increase their commercial value (22, 23).

Bee bread is a by-product of bees, made by adding nectar and bee saliva enzymes to pollen collected by bees before it goes through the process of lactic acid fermentation in the hives (24). Growing consumer interest in natural products has stimulated in-depth research into the nutritional properties of bee bread. Bee bread is a nutrient-dense substance containing significant amounts of carbohydrates, proteins, and lipids. Additionally, it comprises micronutrients, including phenolic compounds, minerals, vitamins, and essential amino acids. It also has therapeutic properties such as antioxidant, anti-inflammatory, anti-tumor antimicrobial, and anti-hypertensive activities (25, 26).

As a result of the literature review, no persistence was found on the storage of FRAP, total chlorophyll, total phenolics, and ascorbic acid content in kiwi juice added to the bee bread with RSM and ANFIS. This programming aims to optimize the FRAP, total chlorophyll, total phenolics, and ascorbic acid content of kiwi juice supported with bee bread and subjected to thermosonication using RSM and ANFIS. In addition, the learning method is to investigate the physicochemical properties, phenolic compounds, and bioactive compounds found in control kiwi juice (C-KJ), thermal pasteurized kiwi juice (P-KJ) and thermosonication-treated kiwi juice with bee bread (TS-KJB).

## 2 Materials and methods

### 2.1 Materials

Samples of kiwi juice were procured from local producers in Tekirdag, Türkiye, and stored at 4°C until the experiments were conducted. Kiwi was peeled and pureed using a blender (Waring Commercial Blender Model HGB2WTS3, United States). To remove coarse particles and impurities from the kiwi juice, it was filtered through a double layer of sterilized muslin cloth. Before treatments, raw kiwifruit juice was mixed into bee bread (Anatolian origin, characterized by HPLC) using a vortex mixer. The resulting mixture was passed through a sieve and thoroughly mixed using a vortex. The sample subjected to this treatment was referred to as the C-KJ. Before each analysis, all juice samples were homogenized using a vortex mixer to ensure uniform distribution of components.

### 2.2 Methods

#### 2.2.1 Thermosonication processing

TS process was carried out on a 100 mL kiwifruit sample using an ultrasonic device (Hielscher Ultrasonics Model UP200St, Berlin, Germany) operating at 26 kHz frequency and 200 W power. Within the scope of this research, the ultrasound parameters evaluated included amplitude, treatment time, and temperature. Amplitude was tested at 40, 50, 60, 70, and 80%. Treatment time was varied between 2, 4, 6, 8, and 10 min. Temperature values were applied in constant mode at 40, 45, 50, 55, and 60°C. Additionally, bee bread concentrations of 20, 40, 60, 80, and 100 mg/100 mL were used.

To prevent the samples from overheating during ultrasonic treatment, they were cooled in an ice bath. Following the thermosonication (TS) process, the bee bread was added (TS-KJB), rapidly cooled in an ice bath, and stored at −18 ± 1°C until analysis. In all trials, bee bread was added to the kiwi juice prior to the thermosonication process.

#### 2.2.2 Thermal pasteurization

The samples of kiwifruit juice were pasteurized at 85°C for 2 min in a water bath using the following equipment (Wisd model WUC-D06H, Daihan, Korea). P-KJ samples were cooled to 20 ± 1°C and stored at −20 ± 1°C until required.

#### 2.2.3 Thermosonication modeling procedure for RSM and ANFIS

This study aimed to evaluate the effects of bee bread and thermosonication on the bioactive components of kiwifruit juice. Specifically, ferric reducing antioxidant power (FRAP, μmol/100 mL), total chlorophylls (μg/mL), total phenolic content (GAE mg/100 mL), and ascorbic acid content (mg/100 mL) were analyzed.

For this purpose, Response Surface Methodology (RSM) and Adaptive Neuro-Fuzzy Inference System (ANFIS) were employed. The independent variables were bee bread concentration (X_1_: 40–100 mg/100 mL), treatment time (X_2_: 2–10 min), ultrasound amplitude (X_3_: 40–80%), and temperature (X_4_: 40–60°C). The dependent variables were FRAP, total chlorophylls, total phenolics, and ascorbic acid content. Preliminary studies identified critical threshold values for parameter optimization: amplitudes below 40% reduced cavitation efficiency, while values above 80% posed a risk of thermal degradation. Treatment times exceeding 10 min resulted in a decrease in chlorophyll stability, while increasing temperatures above 60°C accelerated this degradation to a statistically significant level (*p* < 0.05). Dose–response analyses of bee bread concentration revealed that 80 mg/100 mL was optimal for maximum phenolic content. These findings formed the experimental basis for the selected parameter ranges.

To optimize the thermosonication process using RSM, a Box–Behnken design was applied through Minitab software (version 19, Minitab Inc., State College, PA, United States). The design generated 31 experimental runs based on four factors, with each experiment run in three times. The results are presented in Table 1. The model’s statistical relevance was examined through variance analysis (ANOVA) with a significance level of *p* < 0.05. Model fit was evaluated using lack-of-fit tests, R^2^, adjusted R^2^, and ANOVA results.

**Table 1:** Results of thermosonication using RSM and ANFIS analysis of dependent and independent parameters, FRAP, total chlorophyll, total phenolics, ascorbic acid.

Run no,	Independent variables				Dependent variables											
	Bee bread (mg/100 mL) (X_1_)	Ultrasound time (min) (X_2_)	Ultrasound amplitude (%) (X_3_)	Temperature (°C) (X_4_)	FRAP (μmol/100 mL)			Total chlorophylls (μg/mL)			Total phenolics (GAE mg/100 mL)			Ascorbic acid (mg/100 mL)		
					Experimental data	ANFIS predicted	RSM predicted	Experimental data	ANFIS predicted	RSM predicted	Experimental data	ANFIS predicted	RSM predicted	Experimental data	ANFIS predicted	RSM predicted
1	40	4	70	55	274.81	271.51	275.82	7.12	7.03	7.06	119.48	119.48	119.85	13.28	13.28	13.23
2	40	8	50	55	278.30	274.96	278.98	6.71	6.63	6.71	121.00	121.00	121.22	13.44	13.45	13.44
3	80	4	50	45	288.90	288.90	289.67	6.32	6.32	6.36	125.61	125.61	125.88	13.96	13.96	14.04
4	80	8	50	55	284.93	284.93	285.53	6.19	6.19	6.21	123.88	123.88	124.07	13.76	13.77	13.75
5	40	4	50	45	294.89	294.89	295.16	6.78	6.78	6.78	128.21	128.21	128.27	14.35	14.35	14.30
6	40	8	50	45	274.41	274.41	274.99	6.49	6.49	6.47	119.31	119.31	119.49	13.26	13.26	13.29
7	60	6	80	50	290.46	290.46	290.20	6.31	6.31	6.34	126.29	126.29	126.11	14.03	14.03	14.02
8	40	8	70	45	262.52	259.37	263.06	6.34	6.26	6.35	114.14	114.14	114.31	12.68	12.68	12.62
9	60	6	60	60	267.30	264.09	267.59	6.06	5.99	6.11	116.22	116.22	116.27	12.91	12.92	12.99
10	80	8	70	45	299.07	299.06	300.35	6.13	6.13	6.15	130.03	130.03	130.52	14.45	14.45	14.57
11	80	8	70	55	304.84	304.84	305.47	6.18	6.18	6.16	132.54	132.54	132.74	14.93	14.93	14.93
12	40	4	50	55	282.35	282.35	281.01	6.62	6.62	6.61	122.76	122.76	122.11	13.64	13.64	13.58
13	60	6	60	50	290.84	290.67	290.88	7.18	7.174	7.18	126.45	126.27	126.40	15.68	15.66	15.68
14	80	4	70	45	294.88	294.88	295.11	6.53	6.53	6.51	128.21	128.21	128.25	14.25	14.25	14.20
15	60	6	60	50	290.40	290.67	290.88	7.18	7.174	7.18	126.26	126.27	126.40	15.68	15.66	15.68
16	60	6	60	50	291.68	290.67	290.88	7.14	7.174	7.18	126.82	126.27	126.40	15.64	15.66	15.68
17	60	2	60	50	274.97	274.97	275.50	7.02	7.02	7.05	119.55	119.56	119.72	13.28	13.29	13.30
18	60	6	60	40	277.55	277.55	276.63	6.32	6.32	6.28	120.67	120.68	120.21	13.41	13.41	13.35
19	60	6	60	50	291.06	290.67	290.88	7.18	7.174	7.18	126.55	126.27	126.40	15.68	15.66	15.68
20	40	4	70	45	280.02	280.02	279.36	7.05	7.05	7.05	121.75	121.75	121.39	13.20	13.20	13.28
21	60	6	60	50	290.10	290.67	290.88	7.18	7.174	7.18	126.13	126.27	126.40	15.68	15.66	15.68
22	80	4	70	55	282.72	282.72	282.09	6.06	6.06	6.09	122.92	121.94	122.58	13.66	13.66	13.69
23	60	6	60	50	291.40	290.67	290.88	7.18	7.17	7.18	126.70	126.27	126.40	15.68	15.66	15.68
24	100	6	60	50	310.18	310.18	309.13	6.02	6.02	5.97	134.86	133.78	134.34	14.98	14.98	14.91
25	80	8	50	45	291.14	291.13	291.03	6.34	6.34	6.39	126.58	125.57	126.47	14.06	13.95	14.06
26	60	10	60	50	279.88	279.88	278.71	6.82	6.82	6.81	121.69	121.69	121.11	13.52	13.41	13.53
27	40	8	70	55	278.50	278.50	277.67	6.81	6.81	6.78	121.09	121.08	120.65	13.45	13.35	13.44
28	20	6	60	50	286.40	286.41	286.82	6.96	6.96	7.02	124.52	124.53	124.63	13.59	13.43	13.68
29	60	6	60	50	290.40	290.67	290.88	7.18	7.17	7.18	126.26	126.27	126.40	15.68	15.66	15.68
30	60	6	40	50	286.44	283.86	286.07	6.14	6.14	6.13	124.54	123.42	124.31	13.84	13.71	13.86
31	80	4	50	55	265.66	263.27	266.03	5.79	5.74	5.76	115.50	114.46	115.60	12.83	12.72	12.85
RSM optimization parameters	82.22	7.17	64.64	49.29	305.41			6.61			132.78			15.68		
Experimental values					312.57 ± 4.34			6.15 ± 0.07			127.97 ± 5.81			14.89 ± 0.42		
% Difference					2.30			6.70			3.62			5.03		
ANFIS optimization parameters	82.20	7.17	64.70	49.29	309			6.44			132			15.4		
Experimental values					312.76 ± 3.79			6.12 ± 0.1			127.54 ± 1.57			14.18 ± 0.471		
% Difference					1.20			4.96			3.15			3.43		

X_1_, bee bread (mg/100 mL); X_2_, ultrasound time (min); X_3_, ultrasound amplitude (%); X_4_, temperature (°C); ANFIS, adaptive neuro-fuzzy inference system; RSM, response surface methodology; FRAP, ferric-reducing antioxidant power.

#### 2.2.4 RSM and ANFIS comparison

To assess the accuracy of the models constructed in this study and the selection of the most appropriate model, performance evaluation metrics (R^2^, MAPE, RMSE) were used. The formulation of each parameter used and the desired values for model validity are summarized in Table 2, adapted from the study by Olatunji et al. (27).

**Table 2:** The statistical defect values are described in the following section.

Parameter	Equation	Desirable value
R^2^	$=1-{\left(\frac{\sum_{i=1}^{N}\;(O_{i}-O)(P_{i}-P)}{\sqrt{\sum_{i=1}^{N}\;{\left(O_{i}-O\right)}^{2}(\sum_{i=1}^{N}\;{\left(P_{i}-P\right)}^{2}}}\right)}^{2}\;$	Close to 1
RMSE	$=\sqrt{\frac{1}{N}\;\sum_{i=1}^{N}\;{\left(O_{i}-P_{i}\right)}^{2}}$	Close to 0
MAPE	$=\frac{1}{N}\;\sum_{i=1}^{N}|\frac{(O_{i}-P_{i}}{O_{i}}|\times 100\%$	Close to 0

RMSE, Root mean square error; R^2^, Correlation coefficient; MAPE, Mean absolute percentage error. Where: N, the number of samples; i, the sample index; O_i_, observed yield for the ith sample; O, average observed values; P_i_, value of the predicted yield for ith sample; P, average predicted values.

#### 2.2.5 Ferric-reducing antioxidant power

Thaipong et al. (28) adapted the FRAP assay to assess the total antioxidant activity. The working solution was prepared by mixing 50 mL acetate buffer (0.3 mol L^−1^, pH 3.6), 5 mL 2,4,6-tri (2-pyridyl)-1,3,5-triazine (TPTZ) solution (0.01 mol L^−1^) and 5 mL FeCl₃-6H₂O solution (0.02 mol L^−1^). The solution was stored at 37°C before use. 4.9 mL of the working solution was added to 0.1 mL of the test sample and allowed to react at 37°C for 10 min. The absorbance of the colored product formed was measured at 593 nm. Trolox was used as a standard, and the results were reported in millimoles of Trolox equivalent (TE) per liter.

#### 2.2.6 Total chlorophyll

Hiscox and Israelstam (29) described the method for estimating chlorophyll content. 3 mL of kiwi juice was added to 3 mL of 80% (v/v) acetone. The liquid was filtered three times through Whatman filter paper. The absorbance at 645 nm and 663 nm was measured on the resulting filtrate. The following equations were used to calculate the total chlorophyll content.


(1) Chlorophylla=(11.85×A664)−(1.54×A647)

(2) Chlorophyllb=(21.03×A664)−(5.43×A647)

(3) Total chlorophyll=(chlorophylla)−(chlorophyllb)

#### 2.2.7 Total phenolics

Chromatography was performed by Portu et al. (30) on a C-18 (250 × 4.6 mm; 5 μm packing; Agilent) ACE genix column. The analysis of the polyphenols was carried out on an Agilent 1,260 chromatograph equipped with a DAD. The flow rate was set at 0.80 mL/min. The column temperature was fixed at 30°C. Gradient elution was performed using eluent A and eluent B. Solution A: water with 0.1% phosphoric acid, solution B: acetonitrile. The following gradient was used: 17% B (0 min), 15% (7 min), 20% (20 min), 24% (25 min), 30% (28 min), 40% (30 min), 50% (32 min), 70% (36 min) and 17% (40 min). The injection volume for phenolic analysis was 10 μL. The UV–Vis spectrophotometer was used for the study at 280, 320 and 360 nm. Readings are expressed as μg/mL sample.

#### 2.2.8 Ascorbic acid

The content of ascorbic acid was measured according to the methods of Ordóñez-Santos and Vázquez-Riascos (31). 0.2 grams of oxalic acid (C_2_H_2_O_4_) was added to 30 milliliters of kiwifruit juice. A permanent dark purple color was obtained by titrating 10 mL of the solution with 2,6-dichloroindophenol (DPIP) reagent. Equation (4) was used to calculate the concentration of ascorbic acid.


(4) $$\begin{array}{l} \text{Ascorbic acid}\;(\mathrm{mg}/100\mathrm{ml})=\mathrm{MVC}\times \text{MDPIP}\times \\ \text{VDPIP}∕10\times \mathrm{VS}\; \end{array}$$

CDPIP = molar concentration (mol/L) of DPIP, *M_VC_* = molar mass (g/mol) of ascorbic acid, VDPIP = volume of DPIP (l), VS = sample volume (l).

#### 2.2.9 Analysis of physicochemical

pH measurements were carried out at 20°C using a potentiometer (Hanna Instruments HI 2002 pH/ORP, Romania). The soluble solids content was determined using a refractometer (ATAGO RX-7000α, Japan), and the results were expressed as °Brix value (32). Centrifugation at 5,000 × g for 20 min at 4°C was performed on a 6 mL juice sample from each treatment. The supernatant was collected and 2 mL distilled water was added to mix. The resulting mixture’s absorbance was immediately measured at 660 nm using a spectrophotometer (UV-5200PC, Shanghai, China) with distilled water as a blank (33).

#### 2.2.10 Dietary fiber

The analysis of dietary fiber was carried out by the total filter bag method using an ANKOM 200 fiber analyzer (34). Fruit juice samples without air were filtered, bagged and sealed. In the first stage, a disintegration process was carried out with sulfuric acid (0.255 N) under heating and disintegration conditions for 40 min, then rinse with water and allow to disintegrate for 5 min. In the second stage, the bags were treated with sodium hydroxide (NaOH) (0.313 N) under heating and disintegration conditions for 40 min, followed by 5 min of rinsing with water. At the end of digestion, the bags were kept in acetone for 5 min and not kept at 102°C for 2 h. The total dietary life was calculated using percentages within the failure cases.

#### 2.2.11 Cloud value determination

The cloud value determination method was based on that used by Wang et al. (33). The determination method was based on that used several modifications were made to the experimental design. Centrifugation at 5,000 × g for 20 min at 4°C was performed on 6 mL of juice samples from each treatment. Then 2 mL supernatant was collected and 2 mL distilled water was added to the mix. The absorbance of the mixture was promptly determined at 660 nm through the utilization of a UV–VIS spectrophotometer (SP-UV/VIS-300SRB, Australia), and distilled water was used as blank.

#### 2.2.12 Optimization and validation study

The parameters for thermosonication were optimized for kiwifruit juice enriched with bee bread. This was done using Response Surface Methodology (RSM) and the Adaptive Neuro-Fuzzy Inference System (ANFIS). The employment of a Box–Behnken experimental design with four independent variables was utilized: bee bread concentration (X₁: 40–100 mg/100 mL), ultrasound duration (X_₂_: 2–10 min), amplitude (X_₃_: 40–80%), and temperature (X_₄_: 40–60°C). The effects of these variables on FRAP, total chlorophyll, total phenolics, and ascorbic acid content were evaluated. Thirty-one experimental runs were performed in triplicate, and data were analyzed using Minitab software (version 19) for RSM modeling. ANFIS was used to improve prediction accuracy by integrating fuzzy logic with neural networks. Optimized conditions were examined, and percentage differences between predicted and observed values were calculated to assess model reliability.

#### 2.2.13 Statistical analysis

All tests were conducted in triplicate, and the resulting data are presented as the mean with standard deviation (SD). The data were analyzed using one-way analysis of variance (ANOVA), with differences between means assessed using Tukey’s mentally significant deviation (HSD) test at a significant value of *p* < 0.05. Statistical analysis was done using SPSS 22.0 software (SPSS Inc., Chicago, IL, United States). Three-dimensional RSM plots were generated using Sigma Plot 12.0 statistical analysis software (Systat Software Inc., San Jose, California, United States).

## 3 Results and discussion

### 3.1 Optimization of FRAP, total chlorophylls, total phenolics, and ascorbic acid

The effect of bee bread, time, amplitude, and temperature independent variables on FRAP (Equation 5), total chlorophyll (Equation 6), total phenolic (Equation 7), and ascorbic acid (Equation 8) properties of kiwi juice are shown in the equations below.


(5) $$\begin{array}{l} \text{FRAP}\;(\mu \mathrm{mol}/100\;\mathrm{mL})=\\ 165.0-1.469\;X_{1}-22.94\;X_{2}-3.613\;X_{3}+13.831\;X_{4}+\\ 0.004438\;X_{1}X_{1}-0.8605\;X_{2}X_{2}-0.00686\;X_{3}X_{3}-\\ 0.18767\;X_{4}X_{4}+0.13462\;X_{1}X_{2}+0.02656\;X_{1}X_{3}-\\ 0.02372\;X_{1}X_{4}+0.0485X_{2}X_{3}+0.4536\;X_{2}X_{4}+\\ 0.05310\;X_{3}X_{4} \end{array}$$

(6) $$\begin{array}{l} \text{Total chlorophylls}\;(\mathrm{\mu g}/\mathrm{mL})=\\ 25.49+0.08671\;X_{1}-0.2133\;X_{2}+0.2790\;X_{3}+\\ 0.9146\;X_{4}-0.000424\;X_{1}X_{1}-0.01549\;X_{2}X_{2}-\\ 0.002352\;X_{3}X_{3}-0.009778\;X_{4}X_{4}+\\ 0.002131\;X_{1}X_{2}-0.000145\;X_{1}X_{3}-\\ 0.001060\;X_{1}X_{4}-0.004763\;X_{2}X_{3}+\\ 0.01053\;X_{2}X_{4}+0.000920\;X_{3}X_{4} \end{array}$$

(7) $$\begin{array}{l} \text{Total phenolics}\;(\mathrm{GAE}\;\mathrm{mg}/100\;\mathrm{mL})=\\ 71.7-0.6386\;X_{1}-9.974\;X_{2}-1.571\;X_{3}+\\ 6.013\;X_{4}+0.001929\;X_{1}X_{1}-0.3741\;X_{2}X_{2}-\\ 0.002982\;X_{3}X_{3}-0.08160\;X_{4}X_{4}+\\ 0.05853\;X_{1}X_{2}+0.011546\;X_{1}X_{3}-\\ 0.01031\;X_{1}X_{4}+0.02109\;X_{2}X_{3}+\\ 0.1972\;X_{2}X_{4}+0.02309\;X_{3}X_{4} \end{array}$$

(8) $$\begin{array}{l} \text{Ascorbic acid}\;(\mathrm{mg}/100\;\mathrm{mL})=\\ -49.00+0.0495\;X_{1}-0.007\;X_{2}+0.2422\;X_{3}+\\ 2.2295\;X_{4}-0.000866\;X_{1}X_{1}-0.14181\;X_{2}X_{2}-\\ 0.004341\;X_{3}X_{3}-0.025106\;X_{4}X_{4}+0.006472\;X_{1}X_{2}+\\ 0.001481\;X_{1}X_{3}-0.001158\;X_{1}X_{4}+0.004328\;X_{2}X_{3}+\\ 0.02179\;X_{2}X_{4}+0.003359\;X_{3}X_{4} \end{array}$$

As shown in Equation (8), there was a linear and positive effect on ascorbic acid levels when kiwi juice was thermally processed for longer. Similarly, Yıkmış et al. (35) found that thermosonication had a linear and positive effect on ascorbic acid value in freshly squeezed pomegranate juice.

According to Table 1, FRAP values change depending on the adjustment of the independent variables. There are slight differences between the experimental results and ANFIS and RSM predictions. According to the optimization results obtained with RSM, the ideal parameters for the highest FRAP (305.41 μmol/100 mL), total phenolic (132.78 GAE mg/100 mL) and ascorbic acid (15.68 mg/100 mL) values were determined as 82.22 mg/100 mL bee bread concentration, 7.17 min ultrasound time, 64.64% amplitude and 49.29°C temperature, respectively. The ANFIS model provided lower error margins (1.20–4.96%) with similar optimization parameters (82.20 mg/100 mL bee bread, 7.17 min, 64.70% amplitude, 49.29°C). Generally, the results predicted by the ANFIS and RSM models are closer to the experimental results. For example, an experimental result of 274.81 μmol/100 mL was predicted as 271.51 μmol/100 mL by ANFIS and 275.82 μmol/100 mL by RSM. These differences show that the RSM model gives more consistent results. Similarly, the FRAP value measured experimentally as 304.84 μmol/100 mL was estimated as 304.84 μmol/100 mL by ANFIS and 305.47 μmol/100 mL by RSM. These differences, on the contrary, indicate that ANFIS is more successful. Nevertheless, we can state that RSM is generally more successful. On the contrary, when we look at the optimization parameters, the ANFIS model performed closer to the experimental results (1.20% difference), but the RSM model also provided reasonable agreement (2.30% difference). This shows that the ANFIS model is more successful in FRAP predictions.

**Figure 1:**
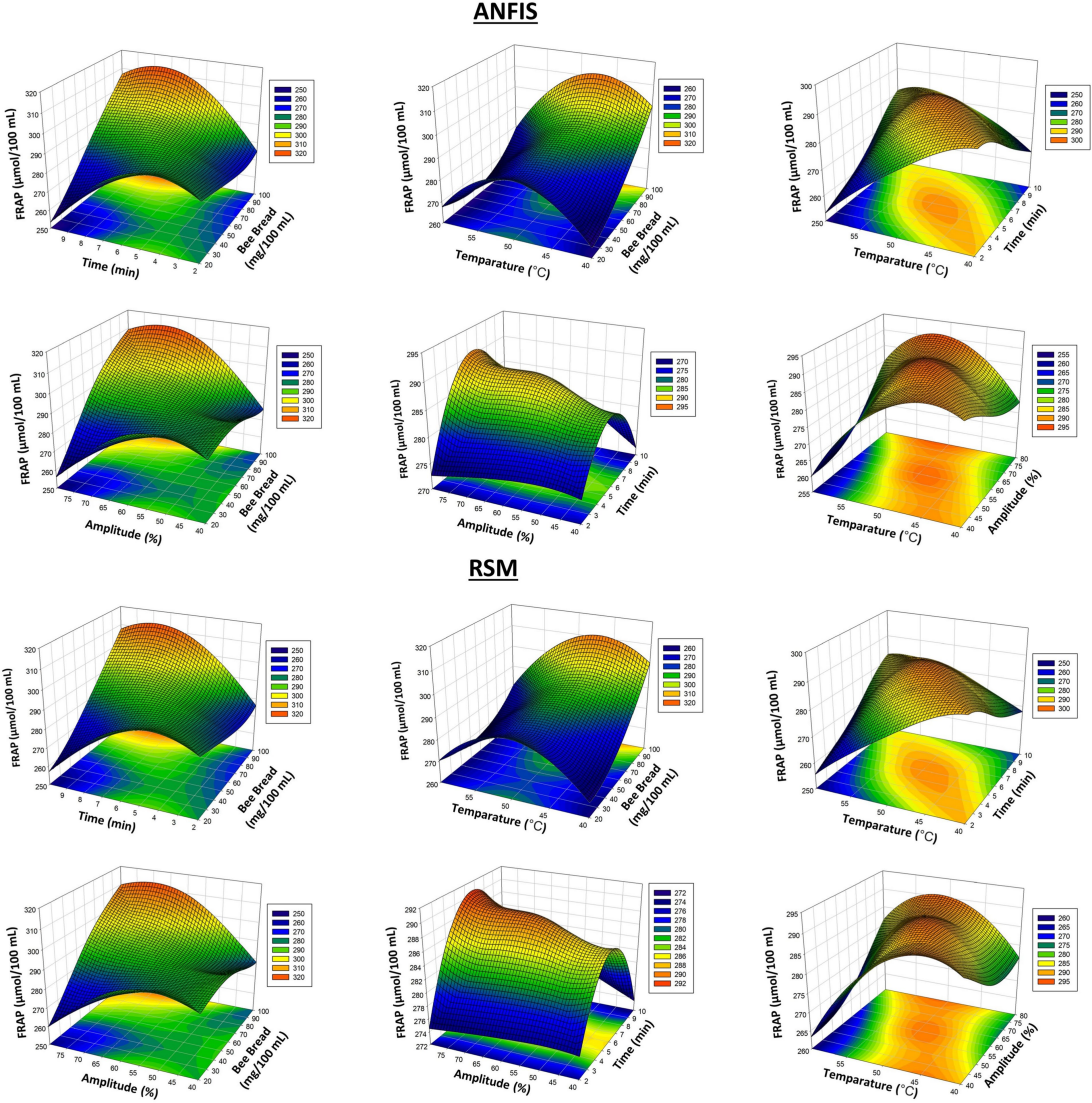
3D plots of FRAP antioxidant results from RSM and ANFIS models.

In this study, ANFIS and RSM models were compared to optimize FRAP, total chlorophyll, total phenolic compounds, and ascorbic acid contents. Figure 1 presents three-dimensional surface plots of FRAP values obtained by ANFIS and RSM models. The findings indicate that the ANFIS model is more effective in predicting FRAP outcomes.

**Figure 2:**
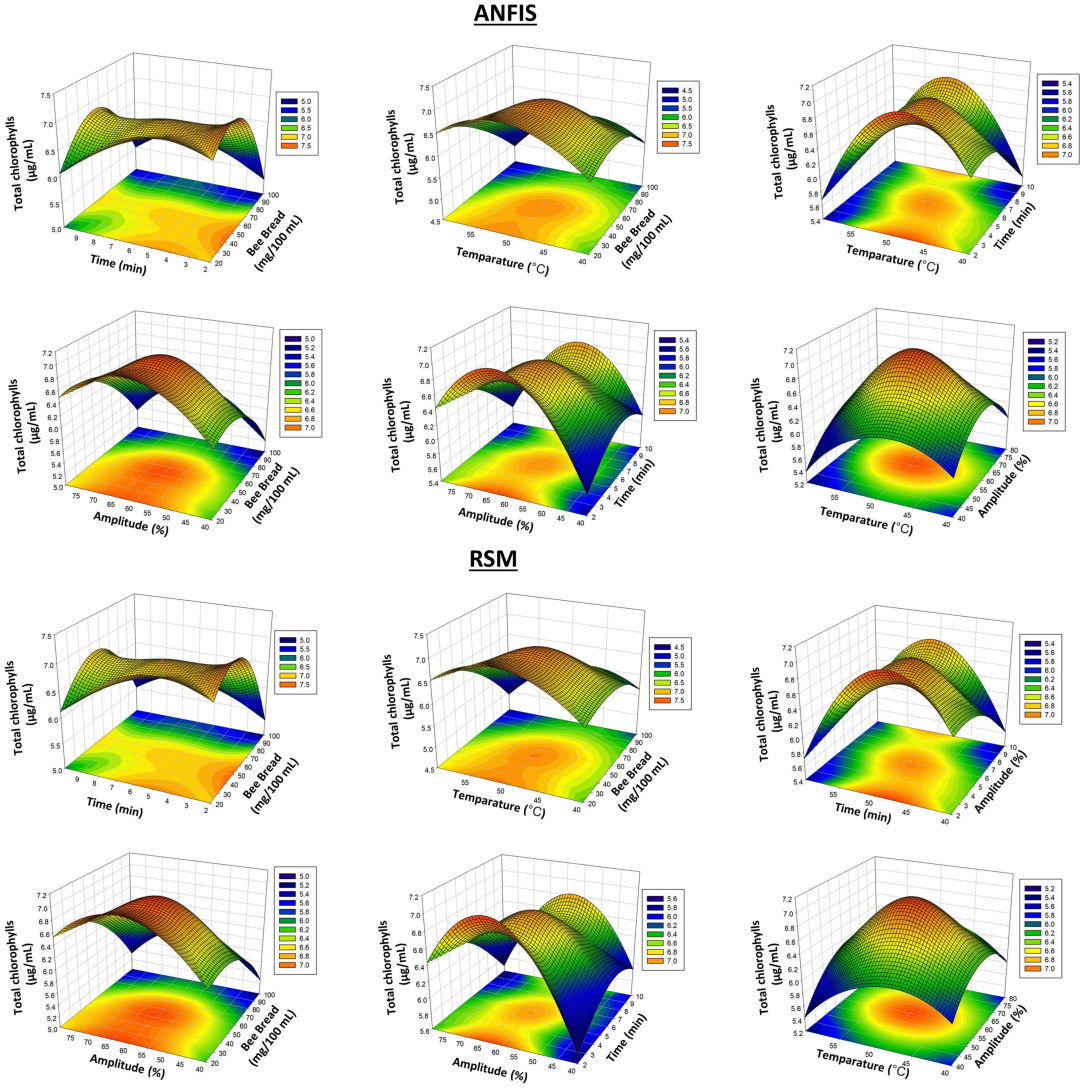
3D plots of total chlorophyll results from RSM and ANFIS models.

Some differences were observed between the experimental results and the models’ predictions. For example, a value measured as 7.12 μg/mL in experimental data was estimated as 7.03 μg/mL by ANFIS and 7.06 μg/mL by RSM. These predictions are quite close to the experimental values, but generally, the ANFIS model produces more accurate results for total chlorophyll contents. In particular, the experimentally measured chlorophyll value of 6.78 μg/mL was estimated as 6.78 μg/mL by ANFIS and 6.78 μg/mL by RSM, indicating that RSM and ANFIS are equally accurate. However, when we look at the optimization differences, it is seen that the ANFIS model is generally more precise, with an error of 4.96%. In Figure 2, the predictions of the ANFIS and RSM models for total chlorophyll contents are compared, and it is observed that the ANFIS model gives more accurate results for this parameter as well.

**Figure 3:**
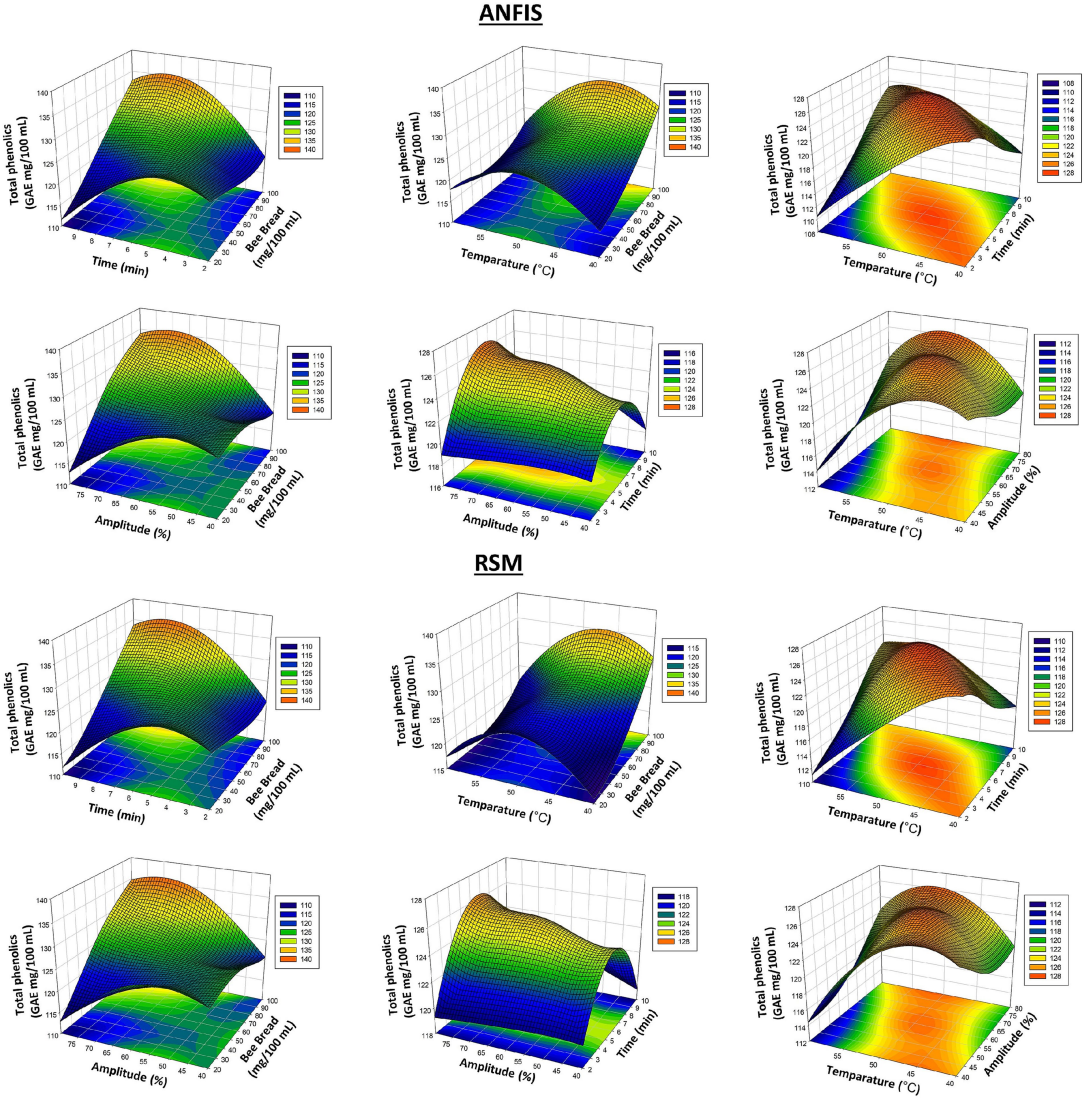
3D plots of total phenolics results from RSM and ANFIS models.

When the table is analyzed, there are significant differences between the experimental phenolic content values and ANFIS and RSM predictions. In particular, a value measured experimentally as 125.61 mg/100 mL is estimated as 125.61 mg/100 mL by ANFIS and 125.88 mg/100 mL by RSM. This indicates that the RSM model has a lower accuracy in phenolic content predictions. In a similar example, the experimental phenolic content of 132.54 mg/100 mL was estimated as 132.54 mg/100 mL by ANFIS and 132.74 mg/100 mL by RSM, indicating a significant underestimation of RSM. When we look at the overall optimization differences, we see that the ANFIS model approached the results with an error margin of 3.15% and the RSM model with an error margin of 3.62%. This indicates that ANFIS is more successful in predicting phenolic content. Ascorbic acid (vitamin C) content was also considered an essential dependent variable in the study. When the ANFIS and RSM predictions were compared with the experimental data, the ANFIS prediction was 14.93 mg/100 mL, and the RSM prediction was 14.93 mg/100 mL, especially for the ascorbic acid content measured experimentally as 14.93 mg/100 mL. Both models have a very high accuracy in ascorbic acid estimation. However, in some cases, the ANFIS model predicted the amount of ascorbic acid more successfully, while in some cases the RSM model predicted it more successfully. When we look at the optimization results, it is seen that the ANFIS model makes more accuracy in ascorbic acid predictions with a margin of error of 3.43%. Figure 3 shows the predictions produced by the ANFIS and RSM models for total phenolic compounds, with the ANFIS model showing more consistent performance on phenolic compounds.

The findings in Table 3 show that the effects on FRAP, total chlorophyll, total phenolics and ascorbic acid were highly significant.

**Table 3:** Corrected *p*-values of linear, interaction and quadratic terms of RSM regression factors of FRAP, total chlorophyll, ascorbic acid, and total phenolics answers as a result of thermosonication.

		FRAP (μmol/100 mL)		Total chlorophylls (μg/mL)		Total phenolics (GAE mg/100 mL)		Ascorbic acid (mg/ 100 mL)	
Source	DF	F-Value	*p*-value	F-Value	*p*-value	F-Value	*p*-value	F-Value	*p*-value
Model	14	266.64	0.000	255.90	0.000	266.64	0.000	483.50	0.000
Linear	4	246.70	0.000	277.90	0.000	246.70	0.000	149.19	0.000
X_1_	1	809.50	0.000	986.46	0.000	809.50	0.000	523.68	0.000
X_2_	1	16.81	0.001	54.90	0.000	16.81	0.001	17.77	0.001
X_3_	1	27.79	0.000	44.22	0.000	27.79	0.000	9,06	0.008
X_4_	1	132.72	0.000	26.01	0.000	132.72	0.000	46.24	0.000
Square	4	290.29	0.000	517.06	0.000	290.29	0.000	1310.85	0.000
X_1_X_1_	1	97.65	0.000	496.72	0.000	97.65	0.000	793.10	0.000
X_2_X_2_	1	367.16	0.000	66.39	0.000	367.16	0.000	2124.98	0.000
X_3_X_3_	1	14.58	0.002	956.67	0.000	14.58	0.002	1244.56	0.000
X_4_X_4_	1	682.27	0.000	1033.72	0.000	682.27	0.000	2601.49	0.000
2-Way Interaction	6	264.17	0.000	67.13	0.000	264.17	0.000	154.82	0.000
X_1_X_2_	1	502.81	0.000	70.36	0.000	502.81	0.000	247.64	0.000
X_1_X_3_	1	489.21	0.000	8.14	0.011	489.21	0.000	324.35	0.000
X_1_X_4_	1	97.57	0.000	108.74	0.000	97.57	0.000	49.58	0.000
X_2_X_3_	1	16.32	0.001	87.83	0.000	16.32	0.001	27.68	0.000
X_2_X_4_	1	356.85	0.000	107.24	0.000	356.85	0.000	175.43	0.000
X_3_X_4_	1	122.25	0.000	20.47	0.000	122.25	0.000	104.24	0.000
Error	16								
Lack-of-Fit	10	3.82	0.057	10.97	0.004	3.82	0.057	29.71	0.000
Pure Error	6								
Total	30								
R^2^		99.57%		99.56%		99.57%		99.76%	
Adj. R^2^		99.20%		99.17%		99.20%		99.56%	
Pred. R^2^		97.80%		97.54%		97.80%		98.66%	

X_1_, Bee Bread (mg/100 mL); X_2_, ultrasound time (min); X_3_, ultrasound amplitude (%); X_4_, Temperature (°C); DF, degrees of freedom; R^2^, coefficient of determination; *p* < 0.05, significant differences; *p* < 0.01, very significant differences.

The overall significance of the model was statistically significant at the *p* < 0.000 level for all response variables (FRAP, total chlorophyll, total phenolics and ascorbic acid). The R^2^ value of the model ranged between 99.57 and 99.76%, indicating that the model explained the data quite well. In addition, the adjusted R^2^ values ranged between 99.17 and 99.56%, indicating that the generalizability of the model is high. In terms of linear effects, X_1_ (bee bread concentration) stands out as the factor with the strongest effect on FRAP, total chlorophyll, total phenolics and ascorbic acid. Especially the *F*-values on FRAP and total phenolics were 809.50 and 809.50, respectively, and were found to be significant at *p* < 0.000 level. Additionally, the effect on ascorbic acid was highly significant (*p* < 0.0001), with an F-value of 523.68 at the *p* < 0.000 level. These findings emphasize the critical importance of bee bread concentration in the extraction of bioactive components. The quadratic effects and two-way interactions of the model also yielded significant results. Among the quadratic effects, X_2_X_2_ (square of ultrasound time) had the highest *F*-values on FRAP, total phenolics, and ascorbic acid (367.16, 367.16, and 2124.98, respectively), indicating that ultrasound duration has a non-linear effect and the best results can be obtained with the optimum duration. The interaction X_1_X_2_ (bee bread concentration and ultrasound time) showed significant effects in the two-way interactions, especially on FRAP and total phenolics. The F-values of these interactions were 502.81 and 502.81, respectively, and were statistically significant at *p* < 0.000 level. In conclusion, factors such as bee bread concentration, ultrasound time, temperature, and ultrasound amplitude play substantial roles in the extraction and stability of bioactive components during the ultrasonic extraction process. The high adjusted R^2^ and predicted R^2^ values of the model support the overall validity and predictive capacity of the model.

**Figure 4:**
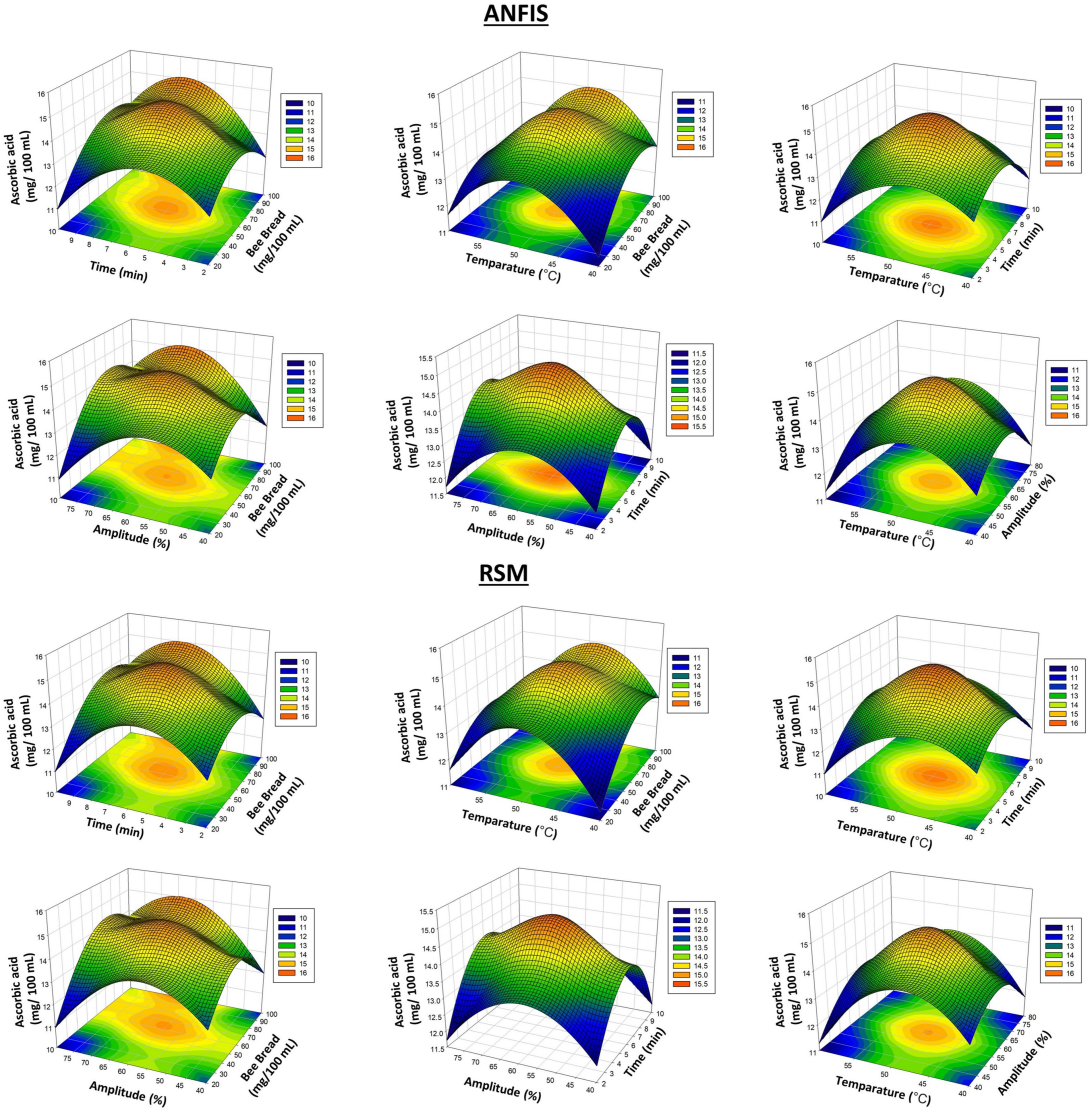
3D plots of ascorbic acids results from RSM and ANFIS models.

In Figure 4, the ascorbic acid results are compared, and it is determined that the RSM model is more successful in predicting ascorbic acid. These graphs show that both models exhibit different performances in specific parameters, and these results are statistically significant. Ascorbic acid (vitamin C) results (Figure 4). Thermosonication process caused an increase in ascorbic acid content. This shows that the process positively affects the preservation of sensitive components such as vitamin C.

### 3.2 Comparison of RSM and ANFIS models

The R^2^ values of the models developed for the estimation of FRAP (μmol/100 mL), total chlorophyll (μg/mL), total phenolics (GAE mg/100 mL) and ascorbic acid (mg/100 mL) ranged between 0.992 and 0.998 (Table 4). All R^2^ values recorded in this study are close to 1, indicating that the models are accurate for these predictions. The RMSE and MAPE values of the models developed for the prediction of FRAP (μmol/100 mL), total chlorophyll (μg/mL), total phenolics (GAE mg/100 mL), and ascorbic acid (mg/100 mL) ranged between 0.03 and 1.354 and between 0.014 and 0.026, respectively. As a result, the RMSE and MAPE values are also within the desired values (i.e., close to 0), and the both RSM and ANFIS results are more satisfactory. In the estimation of all dependent variables except FRAP estimation, the method that gives more satisfactory results varies depending on the RSME and MAPE values. Therefore, the performance of both methods is generally equivalent and successful. According to the results, it is particularly striking that the ANFIS method is more successful in the prediction results depending on the optimization parameters for all independent variables. This result suggests that the ANFIS method may be more successful in intermediate predictions other than the experimental pattern.

**Table 4:** Summary of model performance indices.

Parameters	FRAP (μmol/100 mL)		Total chlorophylls (μg/mL)		Total phenolics (GAE mg/100 mL)		Ascorbic acid (mg/ 100 mL)	
	RSM	ANFIS	RSM	ANFIS	RSM	ANFIS	RSM	ANFIS
R^2^	0.996	0.992	0.996	0.996	0.992	0.993	0.998	0.998
RMSE	0.690	1.354	0.03	0.03	0.305	0.444	0.047	0.054
MAPE	0.016	0.019	0.026	0.017	0.016	0.014	0.019	0.016

R^2^, Correlation coefficient; MAPE, Mean absolute percentage error; RMSE, Root mean square error.

### 3.3 Optimization and validation study

In this study, RSM (Response Surface Methodology) and ANFIS (Adaptive Neuro-Fuzzy Inference System) models were used to optimize the effects of thermosonication on bioactive compounds in kiwi juice enriched with bee bread. Model validation revealed high agreement between experimental results and predictions (R^2^ > 0.992) and low error values (RMSE < 1.354, MAPE < 0.026). These findings demonstrate that both models are reliable for optimizing the selected parameters, but ANFIS performs better, particularly for intermediate-value predictions.

### 3.4 Bioactive compounds

This study comprehensively investigated the effects of different processing strategies on the bioactive contents of bee bread-fortified kiwi juice. Firstly, the dietary fiber content was examined, and the results showed that TS-KJB with bee bread added had the highest dietary fiber content (60.97%). The C-KJ showed a similar value of 59.30%, while P-KJ had the lowest fiber content at 51.72%. These differences were statistically significant (*p* < 0.05), indicating that thermosonication has a protective or even enhancing effect on dietary fiber. Fruit are rich source of antioxidants and total phenolic compounds (36). Kiwifruit fiber can be employed as a natural functional ingredient for encapsulating probiotics or other nutrients, which are then delivered to the gut to facilitate the development of kiwifruit-based functional foods, promoting the enrichment of commensal and probiotic bacteria (37). In a similar study, Nisa et al. (38) found that strawberry juice’s total dietary fiber increased after ultrasonic treatment. Since dietary fiber is critical for digestive health, thermosonication can increase the nutritional value of kiwifruit juice.

Another important finding of the study is that kiwi juice’s antioxidant capacity (FRAP) values vary according to different processing methods. The sample to which thermosonication was applied and bee bread was added (TS-KJB) had the highest antioxidant capacity with 312.57 μmol/100 mL. In comparison, the control group had 283.58 μmol/100 mL, and P-KJ had 268.79 μmol/100 mL. Statistically significant differences (*p* < 0.05) reveal that thermosonication increases the antioxidant capacity. Antioxidants are components that prevent cellular damage and have anti-aging effects. Similar to our study, Sun et al. (39) found that ultrasound-treated kiwi juice samples had significantly higher FRAP values than the control group. These findings show that thermosonication is an effective method in improving the functional properties of kiwi juice. However, when the total chlorophyll contents were examined, it was seen that the processing methods had adverse effects on chlorophyll. The control group (C-KJ) had the highest chlorophyll content with 7.28 μg/mL, while this value decreased to 6.15 μg/mL in the TS-KJB and to 6.02 μg/mL in the P-KJ. Similarly to the present study, Bhutkar et al. (40) found that the ultrasound treatment did not significantly affect the total chlorophyll content compared to fresh kiwi juice. In addition, Zahoor et al. (41) found that total chlorophylls were significantly increased after ultrasound treatment (70% Amplitude, 45°C, and 20 min.) in wheat plantlets juice.

When looking at total flavonoid and total phenolic contents, it was observed that thermosonication process had particularly positive effects on these components. The TS-KJB sample showed the highest value, with 45.15 CE mg/100 mL in total flavonoid content. This value was significantly higher than the flavonoid contents of the control group (C-KJ) with 34.42 CE mg/100 mL and the P-KJ with 28.12 CE mg/100 mL (*p* < 0.05). Flavonoids are known for their strong antioxidant properties and have protective effects against diseases. Similarly, regarding total phenolic contents, the TS-KJB showed the highest value with 127.97 GAE mg/100 mL. The control group had 84.29 GAE mg/100 mL, and the thermally pasteurized sample had 69.45 GAE mg/100 mL. Similar to our study, Wang et al. (42) found that flavonoid compounds of kiwifruit juice increased significantly (*p* < 0.05) with a high-intensity ultrasound process. Total phenolic substances are known for their antioxidant properties and anti-inflammatory effects, and these differences are statistically significant (*p* < 0.01). Similar to our study, Kalsi et al. (43) found that phenolic compounds of guava juice increased significantly (*p* < 0.05) with ultrasound treatment. In a study where Ultrasound-Assisted Aqueous Two-Phase Extraction was developed for the extraction of phenolic compounds from red lotus leaves, TPC and TFC yields increased with increasing temperature and time (44). In contrast with our study, Bhutkar et al. (40) found that ultrasound and heat treatment did not cause any significant change in the phenolic compounds of kiwi juice than to the control sample. Different results may be obtained due to the temperature factor and the addition of bee bread. In addition, when ascorbic acid (vitamin C) content is examined, the TS-KJB sample has the highest ascorbic acid content with 14.89 mg/mL. The control group has 13.25 mg/mL, and the thermal pasteurized sample has 10.38 mg/mL ascorbic acid content. These results show that the increase in ascorbic acid content is statistically significant (*p* < 0.01) and that thermosonication has a protective effect on vitamin C. Similar to our study, Putsakum et al. (9) reported that the 70–100-10 (i.e., temperature-amplitude-time) application resulted in the highest ascorbic acid content (1.10 ± 0.03 mg/100 mL) and was similar to that of the control blackberry juice (1.12 ± 0.07 mg/100 mL). In addition, Sun et al. (39) found that the amount of ascorbic acid was similar in ultrasound-treated kiwi juice samples and control group samples.

**Figure 5:**
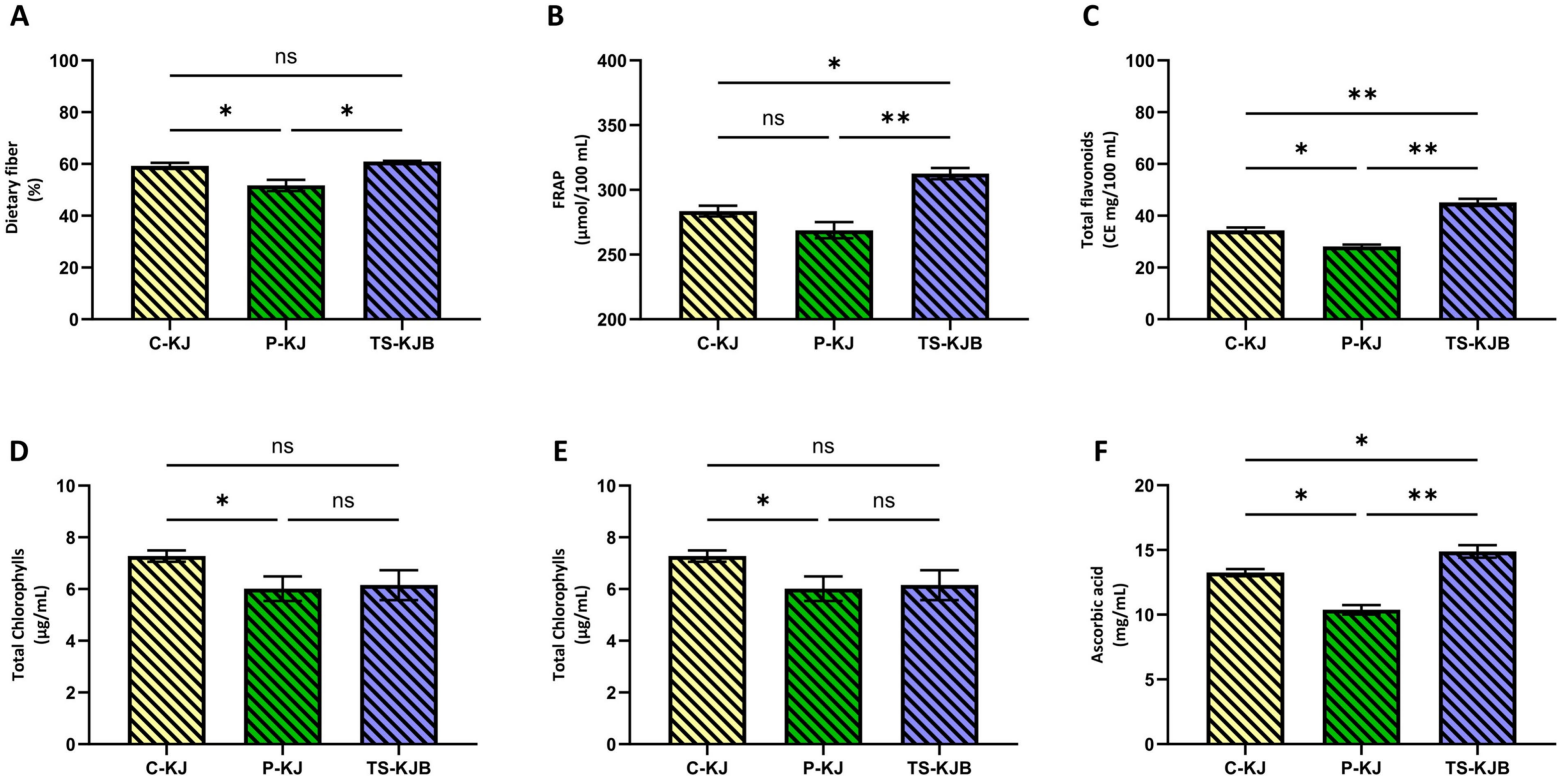
Dietary fiber **(A)**, FRAP **(B)**, Total flavonoids **(C)**, Total phenolics **(D)**, Total chlorophyll **(E)**, Ascorbic acid **(F)**. Characters atop bars indicate statistically significant differences (^*^p < 0.05, ^**^p < 0.01, ^***^p < 0.001). n.s, non-significant; C-KJ, Control kiwi juice; TS-KJB, Thermosonication-treated bee bread-fortified kiwi juice; P-KJ, Thermal pasteurized kiwi juice; ns, not significant.

As a result, the thermosonication process showed positive effects on the bioactive component content of bee bread-fortified kiwi juice. Increased antioxidant capacity, preservation of dietary fiber and phenolic contents, and increased ascorbic acid levels indicate that thermosonication increases bee bread-fortified kiwi juice’s functional and nutritional value. However, a decrease in chlorophyll content reveals that this process negatively affects some pigments. In general, thermosonication can be considered a process that improves the bioactive profile of kiwi juice, especially in terms of phenolic substances and antioxidant components. Bioactive compounds are given in Figure 5.

### 3.5 Physicochemical properties

**Figure 6:**
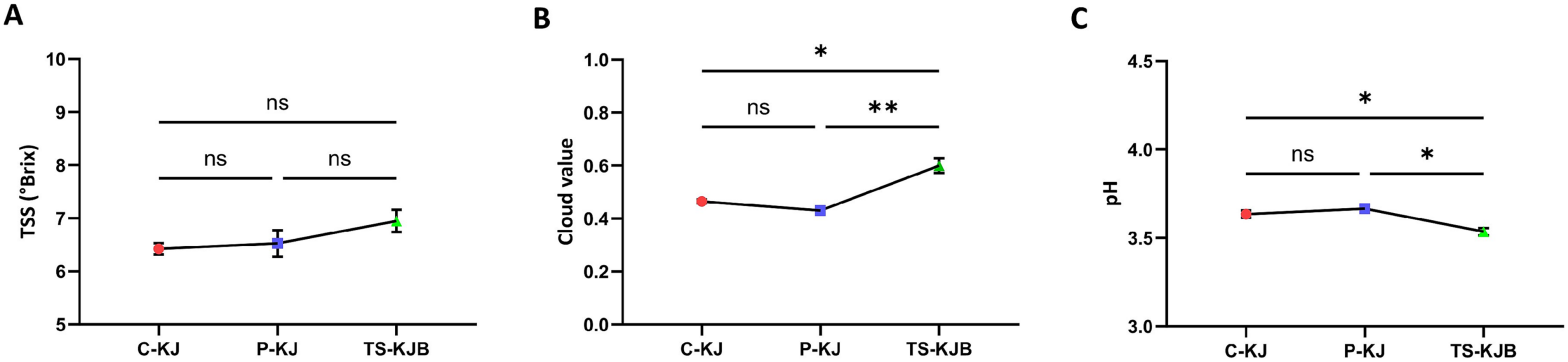
Result for TSS °Brix **(A)**, Cloud value **(B)**, and pH **(C)** of kiwi juices. Characters atop bars indicate statistically significant differences (^*^p < 0.05, ^**^p < 0.01, ^***^p < 0.001). C-KJ, Control kiwi juice; P-KJ, Thermal pasteurized kiwi juice; TS-KJB, Thermosonication-treated bee bread-fortified kiwi juice; ns, not significant.

In this study, the physicochemical properties of kiwi juice obtained through different processing methods were investigated, and the statistical significance of these properties was examined. Three different processing methods were used: control group (C-KJ), thermal pasteurization (P-KJ), and thermosonication supported with bee bread (TS-KJB). The findings were evaluated regarding cloud value, pH, and total soluble solids (TSS) content of the kiwi juice, and the results were supported by statistical analyzes (Figure 6).

Regarding cloud value, the highest value was found in kiwi juice treated with thermosonication supported by bee bread (TS-KJB) at 0.60. This indicates a significant effect of the thermosonication process, which helps keep particles in suspension and increases cloud value. In contrast, thermally pasteurized kiwi juice (P-KJ) had the lowest cloud value (0.43), suggesting that the thermal treatment causes particles to settle. The control group (C-KJ) had a cloud value of 0.47, positioning it between the two extremes. Statistical analyzes revealed that the differences in cloud values were highly significant (*p* < 0.001). In particular, the cloud value of the TS-KJB group showed a statistically significant difference compared to the control and pasteurized groups. This finding demonstrates that the thermosonication process strongly impacts maintaining the stability of suspended particles in kiwi juice, thereby significantly affecting the cloud value. Similarly, Kalsi et al. (10), in their study on guava (*Psidium guajava L*.) juice, concluded that the effect of thermosonication on the cloud value of guava juice was significant compared to that of fresh and pasteurized juice samples (*p* < 0.05), consistent with our findings. The results obtained from these studies are consistent with the strong effect of thermosonication on the cloud value of pomelo (*Citrus maxima*) juice reported by Basumatary et al. (45). This increase has been linked to the process of acoustic cavitation (sonolysis) of macromolecules (such as lipids, cellulose, pectins, hemicellulose, and proteins) present in the juice, during thermosonication, which undergo a series of sonochemical reactions that break them down, thereby enhancing the homogeneity and cloud value of the juice. The observed increase in cloud value following thermosonication is likely attributable to cavitation, providing a high-pressure gradient and enhances the surface area. This process primarily causes the breakdown of pectin and other polysaccharides (such as cellulose and hemicellulose) into smaller molecules (43). Oladunjoye et al. (11), in their study on African mango fruit (*Irvingia gabonensis*), similarly reported that pasteurization (*p* < 0.05) reduced the cloud value level of the juice (from 1.370 ± 0.02 to 1.032 ± 0.03) compared to the control sample. In contrast, thermosonication increased the cloud value level from 1.414 ± 0.01 to 1.687 ± 0.02 with increasing processing conditions. A similar increase in cloud value has also been reported for fruit juices obtained from kiwi and kutkura using thermosonication (12, 33). Additionally, the disruption of tissue cells caused by cavitation leads to the release of intracellular compounds (such as carotenoids and sugars) during the ultrasound process, contributing to the increased cloud value of kiwi juice (33).

The organoleptic quality of fruit juices is determined by pH and TSS (°Brix) (45). The taste of fruit juice is generally determined by a lower pH, contributing to a sour taste. TSS (°Brix) represents the total amount of suspended solids and the concentration of soluble sugars in the juice (12). Regarding pH values, TS-KJB exhibited the lowest pH value of 3.54, indicating that the thermosonication process and the addition of bee bread enhance the acidic nature of the kiwi juice. In contrast, C-KJ and P-KJ had pH values of 3.64 and 3.67, respectively. Although the statistical significance of the differences in pH values was assessed, these differences were found to be non-significant (n.s., non-significant). This result suggests that the pH level of kiwi juice is relatively stable against processing methods, and thermosonication, in particular, does not induce a significant change in acidity. Similarly, Kesavan et al. (12), in their study on kutkura (*Meyna spinosa*) juice, observed pH values of 3.61 ± 0.03 for fresh juice and 3.74 ± 0.04 for thermally processed juice. They concluded that the pH values of the thermosonicated kutkura juices did not exhibit significant alterations compared to pasteurized and fresh kutkura juices. Kalsi et al. (10) reported in their study on guava juice that even after heat and thermosonication treatments, the pH level of guava juice did not change significantly (*p* > 0.05). Similarly, it was shown that both thermosonication and thermal processing did not have a significant effect on the total soluble solids (p > 0.05). Nayak et al. (13) also reported that thermal pasteurization and thermosonication treatments did not have a significant effect on the pH of amora (*Spondias pinnata*) juice samples. These findings can be explained by the fact that the energy levels of ultrasound may not alter the structure associated with the properties mentioned above at a microscopic level (43).

In terms of TSS content, TS-KJB achieved the highest value of 6.95 °Brix. This result indicates that thermosonication increases the concentration of soluble substances (particularly sugars and other organic compounds) in kiwi juice, resulting in a denser texture. In contrast, P-KJ and the C-KJ had TSS values of 6.53 °Brix and 6.43 °Brix, respectively. Statistical analyzes revealed significant differences in TSS values (*p* < 0.05). This finding demonstrates that kiwi juice obtained through thermosonication has a significantly higher TSS content than the other two groups. This increase is thought to result from thermosonication’s high-frequency ultrasonic waves, which make the kiwi juice’s internal structure more conducive to releasing soluble substances. In a study on kutkura (*Meyna spinosa*) juice, similar results were obtained. Fresh, pasteurized, and thermosonicated kutkura juices had total soluble solids of 7.00 ± 0.10 °Brix, 7.00 ± 0.10 °Brix, and 8.00 ± 0.10 °Brix, respectively. It was concluded that pasteurization did not change the TSS of kutkura juice, while thermosonication increased the TSS value (12). In their study, Oladunjoye et al. (14) found that the pH and TSS physicochemical properties of raw hog plum (*Spondias mombin L*.) juice did not differ significantly between pasteurized and thermosonicated samples. Another study found that thermosonication applied to African mango fruit (*Irvingia gabonensis*) juice increased the TSS (°Brix) value compared to the control sample. TSS reflects soluble sugars, so any observed minor changes may be related to the sonolysis of intercellular components, such as fructose, during cavitation (11). The observed increase in the total soluble solids of thermosonicated juices may be associated with alterations in the concentrations of fructose and glucose in these juices, when compared to both pasteurized and fresh juices (15).

Overall, statistical analyzes have revealed that different processing methods applied to kiwi juice significantly affect cloud value and TSS content. Particularly, thermosonication has been statistically validated to have a strong impact on both cloud value and TSS, improving the quality parameters of kiwi juice. These findings suggest that thermosonication could emerge as an innovative method in fruit juice processing and potentially enhance quality in the food industry. In contrast, differences in pH are relatively minor, indicating that the processing methods have limited effects on this parameter. The statistically robust findings provide a significant foundation for further research into the widespread use of technologies like thermosonication in food production.

### 3.6 Determination of individual Phenolics

Determining the chemical composition of kiwifruit is fundamental to demonstrating its nutrition and understanding its potential health effects (46). In many studies examining the effects of kiwi on health, it has been reported that it exhibits many biological activities such as antioxidant, antidiabetic, anti-inflammatory, antihypertensive, anti-asthmatic, anti-carcinogenic, anti-platelet, antifungal, anti-tumor, anti-nociceptive, antiviral, anti-HIV, hepatoprotective, anti-constipation, anti-microbial, and anti-thrombus (47, 48). Among all heat-based preservation methods, pasteurization has been employed for several decades to eradicate pathogens and diminish the proliferation of spoilage microbes in foodstuffs. Although thermal (conventional) methods used for food preservation are applied to extend the shelf life of fruit and vegetable juices through microbial inactivation, their undesirable effects cannot be ignored (16, 49). Consumer demand for juices free of additives and preservatives has encouraged using emerging technologies such as ultrasound, which can deliver higher quality and freshness (50). The thermosonication process, defined as ultrasonically assisted heat treatment, is considered a smart and perfect product technological alternative used to exit the heat treatment used in the processing of fruit juices (17). This study investigated the effects of pasteurization processes and ultrasound processes evaluated within the framework of the “Green Food Processing” concept on phenolic compounds of kiwi juice fortified with bee bread.

The 16 phenolic compounds were quantified in the control kiwi juice, thermal pasteurized kiwi juice, thermosonication-treated kiwi juice with bee bread (Table 5). It was seen that the thermal pasteurization process had a statistically significant negative effect on chlorogenic acid, naringin, rosmarinic acid, salicylic acid, and p-coumaric acid components compared to the control group. Although not statistically significant for phenolic compounds of catechin hydrate, rutin, and quercetin, they showed an increasing trend with pasteurization. This increasing trend may be due to the release of matrix-bound phenolic compounds by heat treatments (51). Heat can inactivate polyphenol oxidase, preventing further loss of phenolic compounds. The increase or decrease in phenolic content depends on the overall composition and types of individual phenolic acids present at maximum in the juice. When phenolic compounds are heated, they tend to undergo some kind of structural rearrangement that can lead to an increase or decrease in antioxidant activities (52).

**Table 5:** Phenolic properties of C-KJ, P-KJ, UT-BJ, and TS-KJB samples.

Phenolic compounds (μg/mL)	Samples		
	C-KJ	P-KJ	TS-KJB
Klorogenic acid	49.89 ± 0.71^b^	37.03 ± 1.52^a^	66.98 ± 0.95^c^
Catechine hydrate	9.38 ± 0.13^b^	9.55 ± 0.40^b^	0.98 ± 0.01^a^
Caffeic acid	3.89 ± 0.05^a^	3.22 ± 0.13^a^	10.22 ± 0.60^b^
4-Hydroxy benzoic acid	0.83 ± 1.17^a^	0.25 ± 0.35^a^	2.84 ± 0.04^a^
Vanillin	6.37 ± 0.09^a^	5.11 ± 0.21^a^	16.43 ± 1.62^b^
p-Coumaric acid	3.09 ± 0.04^b^	2.55 ± 0.11^a^	4.56 ± 0.06^c^
Rutin	1.17 ± 1.65^a^	1.84 ± 0.08^a^	15.69 ± 0.23^b^
t-Ferulic acid	1.55 ± 0.02^a^	0.92 ± 0.04^a^	21.26 ± 0.30^b^
Naringin	19.95 ± 0.29^b^	14.41 ± 0.59^a^	29.24 ± 0.42^c^
Rosmarinic acid	1.51 ± 0.02^b^	0.65 ± 0.03^a^	n.d
Salicylic acid	0.51 ± 0.01^a^	n.d	132.69 ± 11.98^b^
Resveratrol	0.08 ± 0.00^a^	0.01 ± 0.00^a^	13.83 ± 0.2^b^
Quercetin	1.63 ± 0.03^a^	2.93 ± 0.12^a^	92.28 ± 1.32^b^
t-cinnamic acid	0.46 ± 0.01^a^	0.06 ± 0.01^a^	47.33 ± 0.68^b^
Naringenin	0.56 ± 0.01^a^	0.26 ± 0.01^a^	65.84 ± 0.94^b^
Hydroxy cinnamic acid	n.d	n.d	9.39 ± 0.13

Results are presented as mean ± standard deviation (*n* = 3). Values with the different letters within the line are significantly different (*p* < 0.05). C-KJ, Control kiwi juice; P-KJ, Thermal pasteurized kiwi juice; TS-KJB, thermosonication-treated kiwi juice with bee bread; n.d, Not detected showed difference. Values with different letters within the line are significantly different.

Bee bread is a bee pollen-derived fermented product that has attracted attention due to its high nutritional value, especially its phenolic composition, improving quality of life (53, 54). Studies on the phenolic composition of bee bread are limited (55). The most common phenolic compounds (PCs) in bee products are flavonoids and phenolic acids. In general, PCs in bee bread are kaempferol, myricetin, luteolin, isorhamnetin, and quercetin. Gallic acid, protocatechuic acid, caffeic acid, and p-coumaric acid are other phenolics detected in bee bread (53). Sawicki et al. reported the presence of gallic acid, chlorogenic acid, protocatechuic acid and rutin acid in bee bread (56). In the study conducted by Kolayli et al., the phenolic profile of 11 bee bread samples taken from different regions of Anatolian (Türkiye) was examined and high concentrations were detected for t-cinnamic acid, rutin and p-coumaric acid (57). Habryka et al. (58) determined that adding bee bread increased the quercetin level in honey by 20 times. In our study, the amounts of phenolic compounds, except catechine hydrate and rosmarinic acid, increased in bee bread-fortified kiwi juice and subjected to thermosonication. Thermal energy is not the only factor affecting bioactive substances during processing (59). The decrease in the content of bioactive compounds in thermosonicated fruit juices is attributed to oxidation reactions occurring during thermosonication (13). Baltacıoğlu et al., (60) investigated the effect of thermosonication on polyphenol oxidase, peroxidase, and phenolic compounds in apple juice. It was observed that enzyme inactivation was achieved and an increase in phenolic compounds was provided by thermosonication. It is stated in the literature that phenolic compounds are not in free form because they can form hyperlinks with polysaccharides, proteins and cell walls. Bioactive compounds can be released by breaking the cell wall after the TS process. However, it is stated that in some cases, bioactive compounds are degraded by the collapse of microscopic bubbles formed by cavitation and the production of free radicals (61, 62). Phenolic acids and flavonoids may interact synergistically or antagonistically to affect ROS levels (63). The decrease in antioxidant activity affects the degree to which phenolic compounds interact with peroxy radicals (ROO) depending on the presence of hydroxyl groups (OH) (52). In the study of Bayram et al. (64), salicylic acid (19.27–65.20 μg/100 g) was detected as a phenolic compound in bee bread products. According to our findings, the amount of salicylic acid for TS-KJB is, on average, 132.69 ± 11.98 μg/mL, which is higher than the result of C-KJ. It was determined that bee bread-fortified kiwi juice samples and sonication positively affected phenolic content, with some exceptions. Based on these results, Thermosonication can be recommended as a promising method for preserving bioactive forms in fortifying juices with bee bread. With the TS method, high-quality fruit juices can be produced without additives and preservatives by reducing the processing time and time required for the pasteurization of fruit juices.

## 4 Conclusion

This study comprehensively investigated the effects of thermosonication on the preservation and enhancement of bioavailable components of kiwifruit juice fortified with bee bread. The findings indicate that thermosonication is an effective method for the conservation and enhancement of bioactive components such as antioxidant capacity (FRAP), total phenolic content and ascorbic acid. Thermosonication increased the phenolic component content of kiwi fruit juice supplemented with bee bread, while a decrease in total chlorophyll content was observed. This indicates that thermosonication may adversely affect pigment stability and additional research should be carried out to improve these effects. ANFIS and RSM were found to be very successful in predicting bioactive compounds. Furthermore, the ANFIS model was more accurate than the RSM model in predicting dependent variables at optimum conditions. In conclusion, thermosonication process enhances the functional and nutritional properties of kiwi fruit juice enriched with bee bread. It can be considered a sustainable alternative in producing additive-free and preservative-free fruit juice. A limitation of this study is the lack of sensory evaluation of the thermosonicated and bee bread-fortified kiwi juice. Future studies should include sensory analysis to assess consumer acceptance and the impact of processing on organoleptic properties.

## Data Availability

The raw data supporting the conclusions of this article will be made available by the authors, without undue reservation.
